# Statistical analysis plan for HOMESIDE: a randomised controlled trial for home-based family caregiver-delivered music and reading interventions for people living with dementia

**DOI:** 10.1186/s13063-023-07327-8

**Published:** 2023-05-08

**Authors:** Vanessa Pac Soo, Felicity A. Baker, Tanara Vieira Sousa, Helen Odell-Miller, Karette Stensæth, Thomas Wosch, Anna A. Bukowska, Jeanette Tamplin, Nicola Lautenschlager, Sabine Braat, Karen E. Lamb

**Affiliations:** 1 Centre for Epidemiology and Biostatistics, Melbourne School of Population and Global Health, The University of Melbourne, Melbourne, VIC Australia; 2 Methods and Implementation Support for Clinical Health (MISCH) Research Hub, Faculty of Medicine, Dentistry and Health Sciences, The University of Melbourne, Melbourne, VIC Australia; 3 Faculty of Fine Arts and Music, The University of Melbourne, Melbourne, VIC Australia; 4 Centre for Research in Music and Health, Norwegian Academy of Music, Oslo, Norway; 5 Cambridge Institute for Music Therapy Research, Anglia Ruskin University, Cambridge, UK; 6 Hochschule für angewandte Wissenschaften Würzburg-Schweinfurt, Würzburg, Germany; 7 Institute of Applied Sciences, University of Physical Education, Kraków, Poland; 8 Austin Health, Melbourne, VIC Australia; 9 Academic Unit for Psychiatry of Old Age, Department of Psychiatry, Melbourne Medical School, The University of Melbourne, Melbourne, VIC Australia; 10 NorthWestern Mental Health, Melbourne Health, Melbourne, VIC Australia

**Keywords:** Dementia, Music therapy, Statistical analysis plan, Randomised controlled trial

## Abstract

**Background:**

Most people with dementia live in the community, not in residential care. Therefore, quality informal care for them is critical for managing behavioural and psychological symptoms of dementia (BPSD). Music therapy has been shown to reduce BPSD. However, no randomised controlled trial has examined the effects of music interventions delivered by caregivers in home settings. The HOME-based caregiver-delivered music intervention for people living with dementia (HOMESIDE) trial aims to evaluate the effectiveness of a 12-week music intervention in addition to standard care for BPSD. This article describes the statistical analysis plan.

**Methods and analysis:**

HOMESIDE is a large, pragmatic international three-arm parallel-group randomised controlled trial. Dyads (persons with dementia and caregiver) in Australia, Germany, the UK, Poland and Norway were randomised to receive music and standard care, reading and standard care or standard care alone. The primary outcome is BPSD (proxy) of the person living with dementia, measured using the Neuropsychiatric Inventory-Questionnaire (NPI-Q) at 90 and 180 days post-randomisation. Longitudinal analysis will compare NPI-Q severity between music and standard care versus standard care alone. Secondary outcomes include quality of life and depression (both person with dementia and caregiver), cognition (person with dementia only), distress, resilience, competence and caregiver-patient relationship (caregiver only). Treatment effects will be obtained at 90 and 180 days post-randomisation, where applicable. Safety outcomes (adverse events, hospitalisations, deaths) will be summarised.

**Discussion:**

This statistical analysis plan provides a detailed methodology for the analysis of HOMESIDE and will improve the validity of the study and reduce the potential for bias.

**Trial registration:**

Australian New Zealand Clinical Trials Registry ACTRN12618001799246. Registered on November 05, 2018. ClinicalTrials.gov NCT03907748. Registered on April 09, 2019.

## Introduction

The majority of people with dementia live in the community and not in residential care settings [1]. Therefore, personalised care from a family caregiver (CG) is often needed for a person living with dementia (PwD). This personalised care offers benefits to the PwD by enabling them to remain in an environment familiar to them. However, research has shown that managing the behavioural and psychological symptoms of dementia (BPSD) can be difficult for CGs, leading to poor physical and mental health of CGs [2, 3]. This poor health in a CG has the potential to lead to poorer health and well-being for the PwD. Therefore, there is a need to support CGs in providing care.

Music therapy offers the potential to provide CGs with support in managing PwD. This therapy is a registered psychosocial National Health Service intervention in the United Kingdom (UK) to address the needs of people living with dementia [4]. Existing cognitive-behavioural or psychoeducational approaches designed to support CGs have suffered from poor adherence as CGs were unable to attend the scheduled appointments for the intervention [5, 6]. Home-based programmes may be preferable due to their convenience for CGs.

The HOME-based caregiver-delivered music intervention for people living with dementia (HOMESIDE) study is designed to examine the effectiveness of a music intervention (MI) in addition to standard care (SC) delivered directly by CGs in a home setting following training in the intervention delivery by music therapists. The aim of the HOMESIDE randomised controlled trial (RCT) is to demonstrate the effectiveness of the 12-week MI plus SC on the short-term BPSD at the end of the intervention of PwD living at home and being cared for by a cohabitating CG compared with SC (primary) and to evaluate the effectiveness of MI plus SC, compared with a reading intervention (RI) plus SC (secondary). The RI plus SC condition was chosen as an active control group as some evidence suggests reading to and with PwD can have a positive impact on BPSD [7, 8]. The primary BPSD outcome of the trial is the total severity score as measured by the Neuropsychiatric Inventory–Questionnaire (NPI-Q) at 90 days post-randomisation for the PwD. The protocol for the HOMESIDE trial which includes full details of the intervention groups has been published [9].

This paper describes the planned statistical analysis for the HOMESIDE trial and supersedes the plan provided in the trial registries and published protocol. Any changes to the statistical analysis plan between publishing and unmasking will be tracked. The final statistical analysis plan will be approved during the masked data review and signed before breaking the treatment allocation code, after which any changes will be considered post hoc.

## Methods and design

### Setting

HOMESIDE originally planned to recruit participants from six sites in five countries: Australia (Queensland and Victoria), Germany, Norway, Poland and the UK. Initially, training between music therapists and CGs, as well as outcome assessments for all participants, was planned to take place in person. Therefore, participants in Australia were originally only going to be recruited from the states of Queensland and Victoria due to the geographical size of the country. To facilitate the in-person delivery of the intervention across geographically diverse regions, randomisation within Australia was stratified to these two states. Due to the outbreak of coronavirus disease (COVID-19) in March 2020, for the safety of participants and the HOMESIDE team, the delivery of all clinical trial activities moved to an online format (via secured video conference software). This provided scope to recruit participants beyond Queensland and Victoria in Australia. Therefore, the Queensland stratum was subsequently redefined as ‘Northern Australia’ to capture participants recruited from New South Wales, Queensland or the Australian Capital Territory. The Victoria stratum was subsequently redefined as ‘Southern Australia’ and covered participants recruited from the rest of Australia (i.e. Victoria, South Australia, Tasmania, Western Australia, Northern Territory).

### Design

**Fig. 1:**
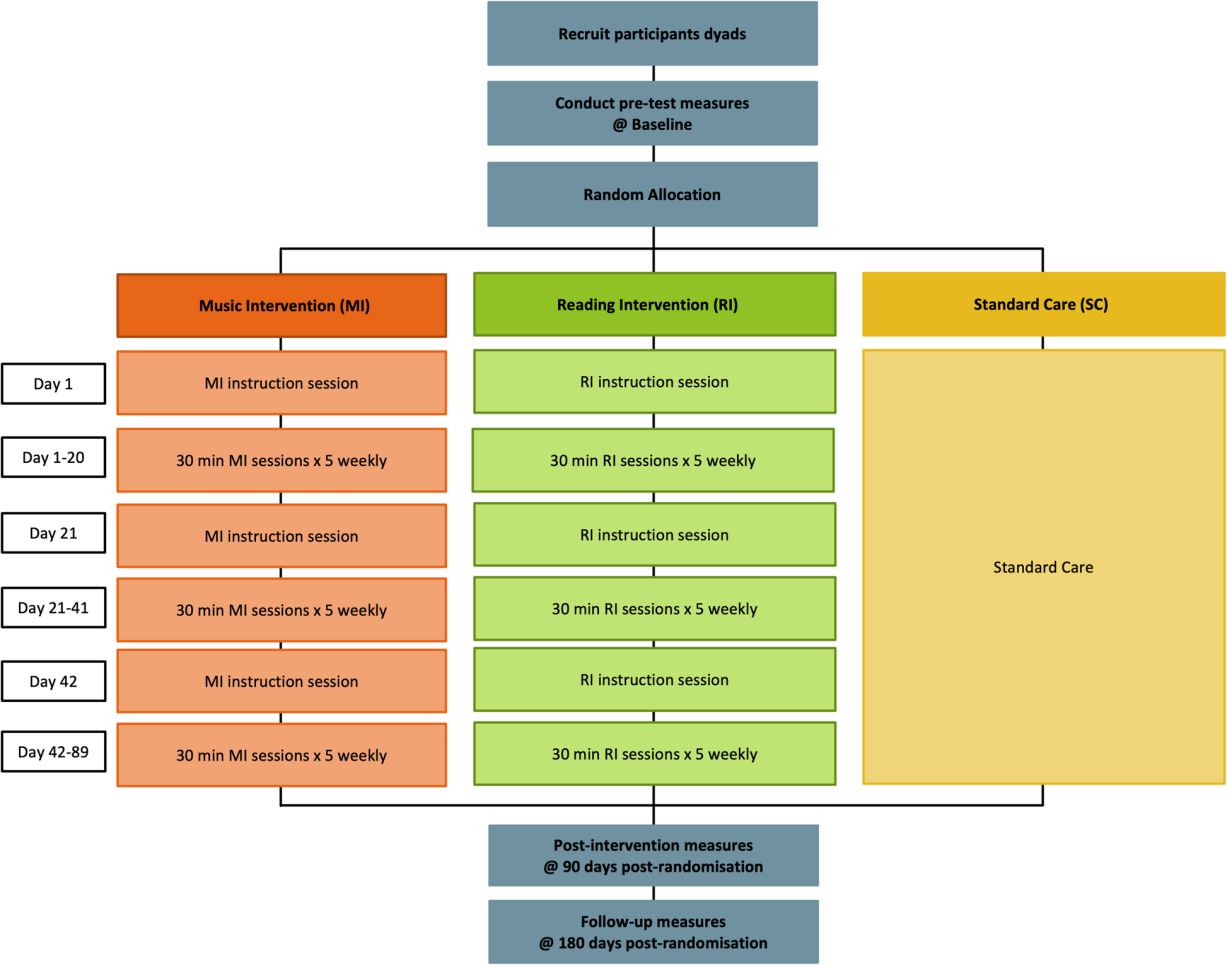
HOMESIDE illustration of study design

HOMESIDE is a large international pragmatic, three-arm parallel-group RCT. Dyads consisting of the PwD and cohabitating CG were randomised to one of the three arms: MI plus SC, RI plus SC or SC alone. HOMESIDE is a superiority trial, with the hypothesis that MI plus SC is superior to SC alone (primary) and RI plus SC (secondary) in relation to BPSD at 90 days post-randomisation. Online assessments occurred at each MI/RI instruction session and follow-up at 90 and 180 days post-randomisation (Fig. 1). The study was prospectively registered at the Australian New Zealand Clinical Trials Registry (ACTRN12618001799246, registered on 05 November 2018) and ClinicalTrials.gov (NCT03907748, registered on 09 April 2019).

### Intervention groups

The interventions were described fully in the detailed published protocol [9]. In brief, the MI involved a 2-h home-based training session in which a qualified music therapist instructed the CG on methods and strategies for using music to assist with care and connection with the PwD. This training covered activities such as singing or listening to familiar or preferred songs and engaging in dialogues about these with the PwD, movement to music and playing instruments. The RI involved a 2-h home-based RI instruction session delivered by a qualified practitioner to engage the PwD during and following the RI. The training covered the CG reading aloud to the PwD, the PwD reading aloud to the CG, listening to audiobooks, playing word games and discussion of the text. Following training, CGs were instructed to deliver the MI or RI at least five times per week for 30 min over a 12-week period. CGs allocated to SC alone were instructed to care for the PwD in their usual manner.

### Eligibility

Dyads were eligible to participate in the study if they were co-habiting in their own homes (i.e. not supported residential care) and the CG was providing care for the PwD. In addition, one member of the dyad had to have a dementia diagnosis (e.g. Alzheimer’s disease, frontotemporal dementia, vascular dementia, Lewy body disease, mixed dementia) and have a score of at least 6 on the NPI-Q. Dyads were not eligible to participate if either or both the CG and PwD had a significant hearing impairment that was not resolved with a hearing aid device as this could limit their capacity to enjoy musical experiences. In addition, participants with no internet access were also excluded from the study. Full recruitment details can be found in the published study protocol [9].

### Randomisation

Dyads were randomly allocated to one of the three intervention groups with a 1:1:1 allocation using a computer-generated schedule of randomly permuted blocks, stratified by site (Queensland [Australia], Victoria [Australia], Germany, Norway, Poland and the UK) to achieve a balance between the arms within each stratum. The list was created by an independent statistician and uploaded to the randomisation module of the REDCap (Research Electronic Data Capture) trial database hosted at the University of Melbourne [10]. The study aimed to have equal numbers of participants within each country. However, due to recruitment challenges at some sites, other sites increased their recruitment target to maximise recruitment. Participating dyads could not be fully blinded due to the active nature of the interventions. The assessors and biostatisticians were blinded to the allocation during the conduct of the trial and will not be unblinded until the database is ready for breaking of the code.

### Sample size

The trial was designed to recruit a total of 495 dyads (165 per arm) to detect a difference of 3 points in NPI-Q total severity score between the MI plus SC and SC alone at 90 days post-randomisation. This assumes 90% power, a two-tailed significance level of 5%, an equal standard deviation of 7.5 points in the groups, no correlation between baseline and 12 weeks (conservative) and 20% attrition. A 3-point change from baseline in NPI-Q total severity score is considered a clinically meaningful difference [11]. No interim analyses to stop the trial early were planned, and no interim analysis was conducted.

### Timing of outcome assessments

The expected visit dates in Table 1 describe the timing of all outcome measures.

**Table 1:** Schedule of enrolment, interventions and assessments

	**Enrolment**	**Allocation**	**Intervention**			**Post-Intervention**	**Follow-up**
**Time point**	***Day − 1***		***Day 1***	***Day 21***	***Day 42***	***Day 90***	***Day 180***
**Enrolment:**							
**Eligibility screen**	X						
**Informed consent (or assent)**	X						
**Dyad: allocation**		X					
**Interventions:**							
***Music intervention training***			X	X	X		
***Reading intervention training***			X	X	X		
**Assessments:**							
**Person with dementia *sociodemographic information***	X						
**MMSE* (self)***	X					X	
**Dementia diagnosis* (proxy)***	X						
**NPI-Q* (proxy)***	X					X	X
**MADRS *(proxy)***	X					X	X
**QoL-AD *(self and proxy)***	X					X	X
**EQ-5D-5L *(self and proxy)***	X					X	X
**Adverse events; death, hospitalisation, death of CG**			X	X	X	X	X
**Caregiver** ***sociodemographic information***	X						
**PHQ-9**	X					X	X
**RS-14**	X					X	X
**SSCQ**	X					X	X
**QCPR**	X					X	X
**AQoL-6D**	X					X	X
**EQ-5D-5L *(self)***	X					X	X
**RUD**	X					X	X
**Post-training questionnaires**			X	X	X		
**Interviews**						X	
**Diary (5 × weekly diary entries for MI and RI)**			X	X	X	X	
**Adverse events: death, hospitalisation**			X	X	X	X	X

Brackets denote who the measure represents (i.e. caregiver (CG), person with dementia (PwD) or dyad), although in some cases the CG provides a proxy measure for the PwD

The EQ-5D-5L and RUD are used in the cost-effectiveness analyses and their analyses are not detailed in the statistical analysis plan

*MMSE* Mini-Mental State Examination Score, *NPI-Q* Neuropsychiatric Inventory Questionnaire, *MADRS* Montgomery-Asberg Depression Rating Scale, *QoL-AD* Quality of Life-Alzheimer’s Disease, *EQ-5D-5L* The EuroQol instrument, *PHQ-9* Patient Health Questionnaire, *RS* Resilience Scale, *SSCQ* Short Sense of Competence Questionnaire, *QCPR* Quality of Caregiver-Patient Relationship, *AQoL-6D* Assessment of Quality of Life-6D instrument, *RUD* Resource Utilisation in Dementia, *MI* Music intervention, *RI* Reading intervention

### Statistical principles

The analysis will be conducted by biostatisticians from the Methods and Implementation Support for Clinical and Health (MISCH) Research Hub (University of Melbourne, Australia). After all study data are available and have been cleaned, a blinded review meeting will be held prior to database lock. The final statistical analysis plan will be signed off before breaking the blind. The analysis of the primary outcome will be independently checked by a senior biostatistician at MISCH and any discrepancies between the two analyses will be discussed and resolved by consensus. All statistical tests will be performed in Stata/SE version 16.1 (Stata Corporation, College Station, TX, USA), and confidence intervals (CIs) reported, at the two-sided 5% level of significance unless stated otherwise.

### Multiple testing

There are a total of three treatment comparisons: (1) MI plus SC vs SC only (2) RI plus SC vs SC only and (3) MI plus SC vs RI plus SC. The comparison of MI plus SC vs SC only and RI plus SC vs SC only will occur at the 5% level of significance. The comparison of MI plus SC vs SC only is the primary comparison. A hierarchical fixed sequence testing procedure will allow for comparison of the primary outcome (NPI-Q severity total score) between MI and RI at 90 days at the 5% significance level if there is strong evidence (*p* < 0.05) of a treatment effect for this outcome comparing MI plus SC and SC only at 90 days.

Secondary outcomes were not powered for. Therefore, no multiplicity adjustment for the analysis of the secondary outcomes will be adopted. Instead, all effect sizes, CIs and *p*-values will be reported to let readers use their own judgement about the relative weight of the conclusions on the effect of the interventions on the secondary outcomes. This approach aligns with the usage of *p*-values favoured by the American Statistical Association [12]. This approach will also be used for exploratory subgroup analyses.

### Adherence

As HOMESIDE dyads live complex lives, it is unlikely that all dyads will adhere to the 12-week intervention. Adherence to the MI/RI intervention will be measured first through CG completed diaries, collecting the number of sessions and the time per session per week. If a dyad supplies a range (e.g. 20–30 min) for the time in a diary entry, the lower limit (i.e. 20 min) will be used for the duration of that day. For dyads with incomplete or missing entries in the diary, we will use the information from the phone call guidelines to measure adherence to the intervention, collecting how often they have been using the programme at each phone call in weeks 2, 5, 7, 9 and 11.

At any given week during the intervention, dyads are deemed adherent if they have reported a minimum of 2 sessions in the completed diaries across the intervention period (i.e. 7 to 90 days post-randomisation). The first week will not be included in the calculation of adherence as it includes the first training session, which may have been held at the end of the week for some dyads. Adherence to the intervention will be derived by summing the number of weeks dyads are deemed adherent across the intervention period. Dyads will be deemed adherent if they scored at least 10 (i.e. a minimum of 10 weeks in which they adhered) and did not have more than two consecutive weeks in which they do not report any engagement with the programme. For dyads who may not be adherent due to incomplete or missing information in the diary, we will use the data captured in the phone call guidelines and consider the dyad adherent if they answered that they have been using the programme 2 days per week or more at all 5 weeks.

Information on the current use of music and reading in daily life at follow-up at 90 and 180 days post-randomisation, irrespective of the randomised intervention, will be summarised by treatment group.

### Analysis populations

The analysis set will consist of all dyads who were randomised, with all dyads reported and analysed according to their randomised study arm. The safety population will consist of all dyads receiving at least one study treatment (including SC alone), with all dyads reported and analysed according to the intervention received. Participants in the MI or RI arms will be considered as having received the study treatment if the dyad attended at least one training session and that all training components were delivered (meeting the fidelity criteria).

### Trial population

#### Trial profile

The flow of dyads through the study will be presented in a Consolidated Standards of Reporting Trials (CONSORT) flow diagram, comprising the number of dyads screened, eligible, consented, randomised and received the allocated intervention. The number of dyads as well as the reasons for exclusion, withdrawal and lost to follow-up will also be reported in the CONSORT diagram.

#### Baseline dyad characteristics

Demographic and baseline variables for both PwD and CG will be summarised and presented by each study arm. Dyads will be described with respect to age, sex, site, marital status, highest level of education, current or last job occupation, main source of income and their current use of music and reading in daily life. Other baseline variables include dementia diagnosis, severity of dementia, time of onset dementia, length of time having dementia (PwD only) and CG’s relationship and length of relationship with PwD will also be reported. Categorical data will be summarised using frequencies and percentages. Continuous variables will be summarised using means and standard deviations, or medians and quartiles (25th and 75th percentiles) for asymmetrically distributed continuous variables.

## Analysis

### Outcome variables

#### Person living with dementia

The primary BPSD outcome of the study is the NPI-Q severity of the PwD at 90 days post-randomisation. Secondary outcomes include NPI-Q severity at 180 days post-randomisation, quality of life (measured by the Quality of Life-Alzheimer’s Disease [QoL-AD]) at 90 and 180 days post-randomisation, depression (measured by Montgomery-Asberg Depression Rating Scale [MADRS]) at 90 and 180 days post-randomisation and cognition (measured by Mini-Mental State Examination Score [MMSE]) at 90 days post-randomisation.

#### Caregiver

Outcomes for the CG include distress (measured by NPI-Q distress), quality of life (measured by Assessment of Quality of Life-6D [AQoL-6D] instrument), depression (measured by Patient Health Questionnaire-9 [PHQ-9]), resilience (measured by 14-item Resilience Scale [RS-14]), sense of competence (measured by Short Sense of Competence Questionnaire [SSCQ]) and the quality of caregiver-patient relationship (QCPR questionnaire) at 90 and 180 days post-randomisation.

#### Safety outcomes

Safety outcomes for both PwD and CG include at least one adverse event, at least one related adverse event (defined as likely or very likely occurred due to the intervention), at least one serious adverse event (hospitalisations or death) and at least one non-serious (other) adverse event.

## Effectiveness analysis

### Primary estimand

The estimand for HOMESIDE is defined according to the addendum to the ICH E9 on estimands and sensitivity analysis in clinical trials [13]. HOMESIDE aims to answer the specific research question: does the addition of music therapy to SC in a home setting delivered by a CG to PwD, compared to SC alone over 90 days, improve BPSD of the PwD, as measured by the mean difference in NPI-Q severity, regardless of the post-randomisation (intercurrent) events listed below, while assigning the worst possible NPI-Q score to the PwD who died.

#### Primary estimand attributes

1. Treatment: MI added to SC compared to SC alone (control), allocation by randomisation.
2. Population: Adults with diagnosed dementia who are residing at home with a caregiver (e.g. sibling, spouse, adult child) who is not paid to deliver care. Full details of the inclusion and exclusion criteria are provided in the protocol [9].
3. Variable: NPI-Q severity at 90 days post-randomisation.
4. Intercurrent events: Possible intercurrent events are outlined below and will be handled using the treatment policy strategy except for death of the PwD for which the composite strategy will be used by assigning the worst-possible NPI-Q score (i.e. 36). Where possible, outcome data will be collected after each of these intercurrent events occur. If no data is collected after an intercurrent event, we will assume that the participant’s outcome after the intercurrent event will be similar to that of all other participants (see details in reporting and methods for missing data).
  - A dyad receiving a different treatment to what they were allocated to
  - Relocation of PwD to a care home
  - A dyad no longer cohabitating
  - A change in primary carer
  - Death of either the PwD or primary carer
  - New medication taken by the PwD or CG (any post-baseline that will affect the trial outcome; see section on concurrent medications for details)
  - Hospitalisation of either the PwD or CG
  - Unplanned or planned surgery or dental work for either the PwD or CG
  - COVID-19 without hospitalisation of either the PwD or CG
5. Population-level summary: mean difference in NPI-Q severity between arms (MI + SC and SC alone).

The number and percentage of dyads who had intercurrent events will be described overall and by treatment arm.

### Primary outcome

The estimate corresponding to the primary estimand of the NPI-Q severity of the PwD at 90 days will be obtained from a likelihood-based longitudinal data analysis model, with responses consisting of all scores (baseline, 90 and 180 days post-randomisation) [14]. The model will include factors representing intervention (SC, MI + SC, RI + SC), time, intervention by time interaction with the restriction of a common baseline mean score across interventions and unstructured variance–covariance among repeated measurements. The model will adjust for the stratification variable (site) used during the randomisation and will be referred to as the unadjusted model. Recruitment site includes Northern Australia, Southern Australia, Norway, the UK, Poland and Germany. The model will include the SC alone group as the reference group.

In sensitivity analysis, variables known to be prognostic of NPI-Q severity (i.e. type of dementia, gender of PwD and CG’s relationship with PwD) will be included in the unadjusted longitudinal data analysis model. This model will be referred to as the adjusted model. The treatment effect estimated from the unadjusted and adjusted models will be the mean difference in NPI-Q severity from baseline to 90 days post-randomisation between the MI plus SC versus SC alone groups.

### Secondary outcomes

Secondary outcomes for the PwD and CG will be analysed similarly to the primary outcome at 90 days and 180 days (where applicable) post-randomisation. The treatment effect will be obtained unadjusted as the mean difference from baseline to post-randomisation between the groups MI plus SC versus SC alone and MI plus SC versus RI plus SC. Appropriate transformations may be applied to the variables before fitting the model if considered skewed.

### Safety outcomes

The number and percentage of PwD and CGs who experience adverse events will be summarised across the intervention period (i.e. randomisation to 90-days post-randomisation), the follow-up period (i.e. 90-days post-randomisation to 180-days post-randomisation) and study period (i.e. randomisation to 180 days post-randomisation) by each intervention group. The total number of events will be reported for the PwD and CG separately.

### Concurrent medications

The type of medications (analgesics, antipsychotics, antidepressants, anti-dementia drugs, anti-epileptics, anti-Parkinson’s, anxiolytics, corticosteroids, hypnotics and sedatives, other nervous system drugs) that the PwD and CG were taking at baseline, 90 and 180 days post-randomisation and study period will be summarised by each intervention group across the intervention period (i.e. randomisation to 90-days post-randomisation), the follow-up period (i.e. 90-days post-randomisation to 180-days post-randomisation) and study period (i.e. randomisation to 180 days post-randomisation.

### Subgroup analyses

Exploratory subgroup analyses will be performed for the primary outcome, NPI-Q severity at 90 days and 180 days post-randomisation. The following subgroups will be explored: (i) gender of PwD (male, female); (ii) gender of CG (male, female); (iii) type of dementia (neurodegenerative diseases [Alzheimer’s disease, frontotemporal dementia, Lewy body disease], mixed [vascular dementia, mixed dementia], other [other or unknown]); (iv) severity of dementia of PwD at baseline (no cognitive impairment [MMSE score of 24–30], mild cognitive impairment [19–23], moderate cognitive impairment [10–18], severe cognitive impairment [< 10]); (v) time of onset dementia (early [under 65 years old] vs late [≥ 65 years old]); (vi) length of time PwD has had dementia (years); (vii) CG’s relationship to PwD (spouse or partner, child or other [sibling, friend, other]); and (viii) country (Australia [Northern Australia and Southern Australia combined], Norway, the UK, Poland, Germany).

For each subgroup analysis, the subgroup (main effect) and its interaction with the intervention group at each time point will be included in the unadjusted longitudinal data analysis model. This will enable the evaluation of whether the treatment effect differs between subgroup categories. Results of the subgroup analyses will be displayed using forest plots, presenting the estimate and two-sided 95% CI of the treatment effect within each subgroup level and the *p*-value of the interaction test. For the length of time that the PwD has had dementia, we will present the between-group difference in the mean change in NPI-Q severity visually, including the 95% confidence intervals.

### Reporting and methods for missing data

Most outcome variables are derived from combining multiple items from a questionnaire to create a score. Therefore, if one or more of the items within the score has a missing response, this affects the derivation of a score for the participant. The number of items missing within an outcome variable will be examined and reported. Decisions on how to handle the missing data will be based on recommended practice for deriving these outcomes, where available. According to recommended practice, if there are two or less items with missing responses in the QoL-AD, MADRS and PHQ-9 instruments, they should be replaced with the mean of the participant’s responses across the complete items [15–17]. Similarly, this replacement will be done by dimension in the AQoL-6D instrument if there is one item with a missing response for that dimension [18]. If there are four or less items with missing responses in the MMSE questionnaire, they should be replaced with the mean of the participant’s responses across the complete items [19]. For all other outcomes combining questionnaire items, if a participant has a missing response to one or more item needed to derive the outcome, the participant will be classified as having a missing outcome.

Following the handling of missing data in outcomes (described above), the primary strategy to handle missing continuous study outcomes will be a likelihood-based approach (i.e. constrained longitudinal data analysis). This approach assumes that the probability of missing data on the study variable is not related to the missing data but to some of the observed measured data in the model (missing at random).

### Additional analyses

It is expected that not all dyads will be adhering to the intervention; therefore, the analysis undertaken for the primary estimand will only provide an estimate of the causal effect of intervention assignment rather than an estimate of the causal effect of the intervention actually received. In a supplementary analysis, the estimate corresponding to a secondary estimand will be obtained that will apply to those dyads who would adhere under any of the three interventions rather than all randomised dyads [20]. The number and percentage of dyads who adhered to treatment will be described. A complier average causal effect (CACE) analysis including all randomised dyads will be undertaken to estimate the average effect of MI plus SC compared to SC on the primary outcome for dyads who would adhere to whichever intervention group they were assigned to, considering a binary definition of adherence. Estimation of the CACE will use an instrumental variable approach in which the randomised intervention group is used as the instrument [21]. The number and percentage of dyads who adhered to treatment will be described overall and by treatment arm.

## Conclusion

The HOMESIDE trial is the first randomised controlled trial to examine a home-based music intervention delivered by a caregiver to manage behavioural and psychological symptoms for people living with dementia. It is an international multi-country study, increasing the generalisability of the findings. The intervention training and assessment were transferred to be online due to the COVID-19 pandemic, restricting participation only to those with internet access. The development of a detailed statistical analysis plan prior to unmasking will reduce the potential for bias and improves transparency.

## Trial status

The trial recruited the first dyad on 26 November 2019, recruitment was completed on 7 July 2022 and follow-up was completed on 31 December 2022. This statistical analysis plan is a detailed plan based on the HOMESIDE protocol [9]. The final statistical analysis plan will be approved and signed before breaking the treatment allocation code, after which any changes will be considered post hoc.

## Data Availability

Not applicable.

## Abbreviations

CG: Caregiver
PwD: Person with dementia
BPSD: Behavioural and psychological symptoms of dementia
UK: United Kingdom
HOMESIDE: HOME-based caregiver-delivered music intervention for people living with dementia
MI: Music intervention
RCT: Randomised controlled trial
SC: Standard care
RI: Reading intervention
NPI-Q: Neuropsychiatric Inventory–Questionnaire
MISCH: Methods and Implementation Support for Clinical and Health
CI: Confidence interval
CONSORT: Consolidated Standards of Reporting Trials
QoL-AD: Quality of Life-Alzheimer’s Disease
MADRS: Montgomery-Asberg Depression Rating Scale
MMSE: Mini-Mental State Examination
AQoL-6D: Assessment of Quality of Life-6D
PHQ-9: Patient Health Questionnaire-9
RS-14: 14-Item Resilience Scale
SSCQ: Short Sense of Competence Questionnaire
QCPR: Quality of caregiver-patient relationship
CACE: Complier Average Causal Effect
